# Practice of clean intermittent catheterisation in children with spina bifida: A scoping review

**DOI:** 10.4102/ajod.v13i0.1473

**Published:** 2024-11-22

**Authors:** Denis Nono, Andrew S. Ssemata, Femke Bannink Mbazzi, Janet Seeley

**Affiliations:** 1 Department of Global Health and Development, Faculty of Public Health and Policy, London School of Hygiene and Tropical Medicine, United Kingdom; 2 Medical Research Council (MRC), Uganda Virus Research Institute (UVRI) and London School of Hygiene and Tropical Medicine (LSHTM) Uganda Research Unit, Entebbe, Uganda; 3 International Centre for Evidence in Disability, Department of Epidemiology and Population Health, London School of Hygiene and Tropical Medicine, United Kingdom; 4 African Health Research Institute, Durban, South Africa

**Keywords:** spina bifida, clean intermittent catheterisation, barriers, facilitators, Africa

## Abstract

**Background:**

Spina bifida is a congenital neural tube defect, where there is incomplete formation of the spinal cord and vertebrae, resulting in abnormal development of the neural tube. This affects bladder function and urinary incontinence. Clean Intermittent Catheterization (CIC) is used to manage bladder and bowel management.

**Objectives:**

This study aims to scope evidence on the facilitators and barriers to usage and practice of CIC in children with spina bifida in low-income countries.

**Method:**

We searched databases including PubMed, Web of Science and SCOPUS, and screened articles for inclusion following the PRISMA-ScR guidelines. The search terms included ‘Spina Bifida ([continence management] AND [clean intermittent catheterisation]) AND ([barriers to Clean Intermittent Catheterisation] OR [Low Income Countries]) OR (myelomeningocele)’. Full-text assessment for eligibility excluded 202 articles. Twenty-two articles were reviewed and twelve full-text articles were excluded because of limited content. Ten articles published in English between 2004 and 2023 were selected for review.

**Results:**

Barriers in practicing CIC include pain and discomfort in catheter insertion, stigma and fears; inaccessibility of public toilets, unavailability of appropriate catheters, difficulty in positioning, limited quality of teaching and challenges with accessing supplies. Facilitators include starting CIC in infancy, follow-up by healthcare providers, support from family and community members, quality of training, continuous practice of CIC, utilisation of lubricants, reuse of catheters and other low-cost materials.

**Conclusion:**

Our review summarises facilitators and barriers to CIC and provides recommendations for further research, which includes the involvement of family members and community-based rehabilitation workers.

**Contribution:**

This article contributes to a better understanding of CIC use in low-income countries.

## Background

The focus of this scoping review is facilitators and barriers to using and practicing catheterisation in persons with spina bifida in low- and middle-income countries (LMICs). Spina bifida is a neural tube defect (NTD), a congenital disability, whereby the spinal cord and vertebrae do not form completely and the neural tube fails to develop normally (Warf et al. 2011). The condition is caused by a fault in the development of the central nervous system in early pregnancy, normally during the first 25 days (Northrup & Volcik 2000). In low-income countries, 66% of children with spina bifida also develop hydrocephalus (Warf et al. 2009), causing paralysis below the lesion and progressive hydrocephalus. As most children with spina bifida have some degree of paralysis, mobility is affected along with bladder and bowel control (Andren & Grimby 2000; Danielsson et al. 2008; Northrup & Volcik 2000; Verpoorten & Buyse 2008; Warf & Campbell 2008). Children with spina bifida are either able to walk with or without aids or they have to use a wheelchair depending on the height of the lesion. It is also evident that this paralysis causes 90% of urinary and faecal incontinence (Verpoorten & Buyse 2008). Children with spina bifida have a high probability of renal complications, including hydronephrosis and renal failure (Kasabian et al. 1992). Notably, effective bladder management is critical to preventing renal damage and promoting social dryness (Filler et al. 2012).

Clean Intermittent Catheterisation (CIC) was introduced in 1972, by Lapides et al. (2002) in persons with bladder dysfunction and urinary incontinence revolutionising neurogenic bladder management. Clean Intermittent Catheterisation is the most commonly used procedure for persons with spina bifida who require an alternate voiding method (Kessler et al. 2009; Van Achterberg et al. 2008; Wilde, Brasch & Zhang 2011). The ability to identify which infants are at the highest risk of urinary tract deterioration has prompted the initiation of prophylactic therapy (Kasabian et al. 1992) including CIC to avoid high intravesical pressures and the use of medications that relax the external sphincter (Kasabian et al. 1992). The therapy is fundamental in the treatment of patients with alterations in bladder voiding and it contributes to avoiding renal damage and improving urinary continence (Sager et al. 2022). Angermund et al. (2021) considered intermittent catheterisation as the gold standard and an effective and safe measure to allow bladder emptying at regular intervals associated with preventing complications of urinary stasis in children with spina bifida (Angermund et al. 2021). Sager et al. (2022) mentions that, to assist children with spina bifida in emptying their bladder, most of them begin CIC shortly after birth. An early start with CIC promotes greater adherence and adaptation of both the child and the members of the family.

Uptake of CIC is associated with the promotion of social inclusion, boosting self-esteem and independence among children with spina bifida, their parents and caregivers (Assis & Faro 2011; Woodward & Rew 2003). The procedure is invasive, requiring a clean, disposable or reusable catheter inserted into the urethra to empty the bladder of urine by the person with spina bifida or his or her caregiver 4–5 times a day. In all, children with spina bifida and their families select catheters depending on personal preference, cost, portability and how easy it is to use (Sager et al. 2022). As a first-choice treatment for bladder emptying, CIC practice can adequately and safely ensure no residues and infections in children with spina bifida and is a valuable tool for achieving continence (Verpoorten & Buyse 2008). In Uganda, social exclusion as an outcome of not practicing CIC among peers has an impact on the sequence of CIC practice and usage (Bannink Mbazzi et al. 2016). Poorly managed incontinence leads to social exclusion among peers, community and family members (Bannink Mbazzi et al. 2016). In addition, social barriers to care and support leave children feeling isolated, demoralised and unsupported during CIC practice, ultimately affecting their long-term adherence to the intervention (Bannink Mbazzi et al. 2016).

In the Global North, the effectiveness of the CIC technique has been documented (Jeruto, Poenaru & Bransford 2004) but there is limited information on its application in LMIC (Jeruto et al. 2004). Principally, CIC, oxybutynin and chemoprophylaxis (trimethoprim 2 mg/kg once daily) in all newborn patients with spina bifida are started immediately after closure of the back (Verpoorten Buyse 2008). This practice ensures safe pressures in the lower urinary tract in most of the patients with spina bifida at that age. In doing so, bladder volume and compliance remain satisfactory for years in most patients (Verpoorten & Buyse 2008). Between the first 8–9 years of life, parents and other caregivers carry out CIC but if the children show sufficient dexterity, they take up the task themselves after this age (Verpoorten & Buyse 2008). After birth, CIC is carried out with self-lubricating 8-F catheters (Verpoorten & Buyse 2008). The size of the catheter depends on the patient’s age; the aim is always to use the largest possible catheter to obtain optimal bladder emptying. Oxybutynin is best started together with CIC immediately after the closure of the back (Verpoorten & Buyse 2008).

Some basic principles encompassing proper education and training, clean and atraumatic application and long-term achievement of good patient compliance are emphasised by rehabilitation practitioners (Verpoorten & Buyse 2008). In East and Southern Africa, long-term outcomes are maintained by ensuring mothers or caregivers are trained on how to keep catheters clean for use and reuse to preserve kidney function (Mertens & Bannink 2012). While practicing CIC, parental care for children aids in the success and achievement of the learning process for children diagnosed with spina bifida (Waugh, Brewer & Wagoner 2022). Not explored in detail are the barriers to practice and usage among children with spina bifida and their families in LMIC settings.

In this review, a summary of the evidence on the facilitators and barriers to the implementation of CIC in children living with spina bifida is set out, specifically focusing on facilitators of usage, and the barriers affecting usage and practice of CIC in LMIC. The specific objectives are:

- to explore the barriers to the implementation of CIC;
- to explore factors that facilitate the usage and practice of CIC; and
- to identify possible recommendations for the implementation of CIC in children with spina bifida in LMICs.

## Research methods and design

The literature search focused on studies conducted between January 2004 and September 2023. The search was conducted in September 2023. Following the scoping review strategy theorised by Joanna Briggs Institute (JBI) in the 1990s, we explored the types of available evidence in a given field, clarified concepts in literature, examined how research is conducted on a certain topic, analysed knowledge gaps and identified key characteristics or factors related to a certain concept (Munn et al. 2018). The JBI strategy we focused on included identifying the research question and relevant studies, selection, data charting, collation, summary and reporting of results, analysis of evidence, presentation of results, summary of evidence, reviewing and conclusion on the barriers and facilitators to CIC in children with spina bifida and their families.

### Search strategy

In accordance with this strategy, potentially eligible articles were identified through PubMed, Web of Science and SCOPUS databases. Additional searches were conducted through Google Scholar and Research Gate. The focus was on LMICs as defined by the Development Assistance Committee (DAC) list of Official Development Assistance (ODA) reporting (OECD 2022). The key search terms were: (‘Spina bifida’)AND (‘Clean Intermittent Catheterisation (CIC)’) AND (‘LMICs’) OR (barriers to Clean Intermittent Catheterization). A repeated advanced query search algorithm derived from the main themes: (‘Spina bifida’) AND (‘continence management’) AND (‘kidney saving’) OR (‘clean intermittent catheterization’) OR (barriers to implementation of Clean Intermittent Catheterization) generated 1995 search results fed separately across the different databases. The key search terms were ‘spina bifida’, ‘Clean Intermittent Catheterisation’, ‘barriers to Clean Intermittent Catheterisation’ and ‘facilitators to Clean Intermittent Catheterisation’. When filtered with the search terms ‘spina bifida’ and myelomeningocele, continence management and barriers to continence management, LMICs yielded 1,150 results. We reviewed both published and grey literature from the International Federation of Spina Bifida and Hydrocephalus (IFSBH) website and other sources focusing broadly on continence management in children with spina bifida and barriers to implementation of CIC. Three additional articles were included from grey literature (IFSBH website). After the removal of duplicates, 223 results were included for title and abstract reviews. Based on the abstracts and titles, we conducted a full-text assessment for eligibility and excluded 201 articles because they focused on spina bifida although without any mention of the barriers or facilitators of CIC, resulting in 22 articles remaining for review. Of the 22 articles, 12 full-text articles were excluded because of limited content in terms of barriers and facilitators to CIC usage and practice. A total of 10 articles, from studies conducted in six countries, were included in the qualitative synthesis. The final studies, a total of 10 articles that were conducted in six countries were included in the qualitative synthesis.

### Information sources and study selection

The review process entailed gathering evidence-based information from primary data sources. The databases included PubMed, Embase, Scopus and Google Scholar. An excel sheet depicting search results was developed followed by logical identification and screening processes to indicate the criteria in terms of relevance for inclusion in this review process.

### Study eligibility criteria

The scoping review included scientific literature based on the nature of the study topic and thematic areas of facilitators and barriers of CIC. The study topics were narrowed to the practice of CIC in children with spina bifida, their families and caregivers. The focus of inclusion was also primarily on the barriers and facilitators of usage and practice of CIC in LMICs. The review excluded grey literature from articles published in non-commercial form including theses, dissertations and government Ministry of Health reports, fact sheets and pre-prints of articles on spina bifida. Only sources written in English focusing on the barriers and facilitators of the usage and practice of CIC were included.

### Data charting and abstraction

Data were populated into an Excel spreadsheet and analysed before extracting and summarising the data. The data abstraction table included the title of the abstract, author information, sample size and demographics and comments on the reviewed articles. The selected articles were checked, and approved by all authors, before inclusion. The Preferred Reporting Items for Systematic Reviews and Meta-Analysis extension for Scoping Reviews checklist (PRISMA-ScR) was followed (Page et al. 2021).

### Synthesis, summarising and reporting of findings

The review results were thematically analysed, modulating one or general arguments for organising the review. Interpretation of findings focused on emerging themes and patterns discovered as results were summarised. The synthesis of results connected the information reported by different sources and not just summarising the results. These also involved interpreting different patterns in the results of different studies aimed at organising them into both inductive and deductive themes.

### Presentation of results

**FIGURE 1:**
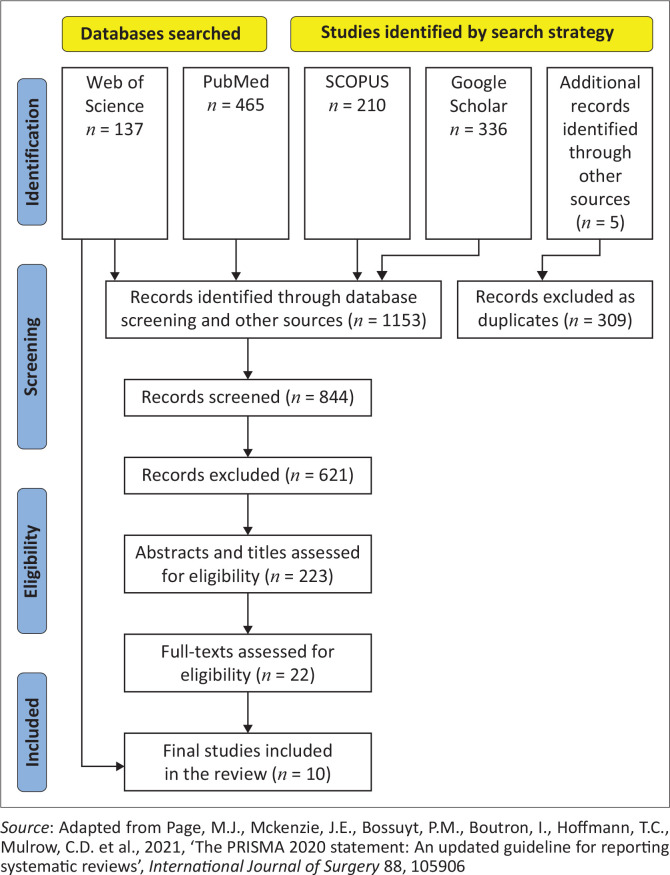
Flow diagram of literature search and screening.

We included studies that provided feedback on barriers and facilitators of CIC among children with spina bifida. We found 1,153 articles focusing on barriers, facilitators and experiences in the usage and practice of CIC in children with spina bifida, their families and caregivers. A total of 309 articles were excluded as duplicates, resulting in 844 articles that were then screened focusing on barriers and facilitators, and a further 621 article records were excluded because the studies lacked content that focused on the themes of the scoping review. After the screening, 223 abstracts and titles were assessed for eligibility. The description of the selection, screening, and inclusion of the studies is presented in Figure 1.

### Study characteristics

The 10 studies were conducted in Brazil (Orlandin et al. 2020), Morocco (Kyal et al. 2018), South Africa (Page & Coetzee, 2021), Uganda (Bannink et al. 2016a, 2016b, 2016c; Bannink Mbazzi et al. 2016; Sims-Williams et al. 2018), Kenya (Jeruto et al. 2004) and Sudan (Ibrahim et al. 2004). The study designs and methods included in the review are scoping review, prospective, descriptive and analytic, explorative qualitative research design, semi-structured interviews using psychometric scales, mixed methods and quasi-experimental.

### Ethical considerations

Ethical clearance to conduct this study was obtained from the Uganda Virus Research Institute Research and Ethics Committee (No. GC/127/985). In addition, research clearance was obtained from the Uganda National Council for Science and Technology.

## Results

This section presents in a thematic manner, the barriers and facilitators to the practice of CIC among children with spina bifida and their caregivers. Several barriers identified in the review inhibit the usage and practice of CIC. The findings covered several themes including pain and discomfort, fear of catheter insertion, a lack of quality training, challenges to self-autonomy and independence in managing toilet activity limited access to medical care, and financial difficulties. Others included a lack of facilities to practice CIC, long and time-consuming process in practicing CIC, difficulty in positioning and the lack of supplies to facilitate the CIC management processes are key barriers. We describe the barriers by theme further in the text.

### Barriers to usage and practice of clean intermittent catheterisation

#### Theme 1: Pain and discomfort

Three studies conducted in Brazil, Morocco and Sudan (Ibrahim et al. 2004; Kyal et al. 2018; Orlandin et al. 2020) reported pain in catheter insertion among both male and female children as one of the main barriers to usage and practice. Orlandin et al. (2020) reporting findings from Brazil noticed that pain is a sensory and unpleasant emotional experience throughout the catheterisation experience in children with spina bifida (Orlandin et al. 2020). Pain inhibits the uptake of usage and practice of CIC because of discomfort (Orlandin et al. 2020). This was further suggested by Lapides et al. (1972) in a study conducted in Korea, the process of emptying the bladder 4–5 times a day was reported as painful and causing discomfort coupled with other challenges of storage, management and irrigation of the catheter.

#### Theme 2: Fear

Linked to pain was fear. Kyal et al. (2018) in their study conducted in Morocco found that the fear of the CIC technique affects long-term adherence, and often patients with spina bifida are afraid of catheters entering their bodies, causing pain and infections. Kyal et al. (2018) expounded that after conducting the first session of therapeutic education to patients and their caregivers with spina bifida, the most pronounced barrier to the uptake of the practice was fear of catheterisation. In Morocco, psychological aspects of the barriers to CIC include being scared of harming children by causing infection or mistakenly breaking their hymen. In terms of practice, there was the fear of mistaking the vagina for urinary meatus (Kyal et al. 2018).

#### Theme 3: A lack of quality training for children, parents, and caregivers

Four studies (Ibrahim et al. 2004; Jeruto et al. 2004; Kyal et al. 2018; Page & Coetzee 2021) described the lack of quality training for children, parents and caregivers as a hindrance to the practice and management of CIC processes. In Morocco, Kyal et al. (2018) found that external factors including limited quality of teaching, and the training environment are significant barriers to the usage and practice of CIC (Ibrahim et al. 2004). In South Africa, Page et al. (2019) reported that the lack of training to accommodate both adolescents and their caregivers on the practice and use of CIC is a significant barrier (Page & Coetzee, 2021). In Sudan, Ibrahim et al. (2004) observed that a lack of proper training encompassing all major steps with hands-on local materials affects the uptake of the practice of CIC (Ibrahim et al. 2004). Reporting findings from Kenya, Jeruto et al. (2004) found that no CIC programme is complete in its follow-up after providing training, and patients are often lost for various reasons (Jeruto et al. 2004).

#### Theme 4: Challenges to self-autonomy and independence in managing toilet activity

Three studies (Bannink et al. 2016b; Kyal et al. 2018; Page & Coetzee 2021) conducted in Morocco, South Africa and Uganda, respectively, described the challenges to self-autonomy and independence in managing toilet activity. In Morocco, Kyal et al. (2018) explored external factors including challenges in accessing public toilets, and the unavailability of appropriate catheters. Furthermore, in South Africa Page and Coetzee (2021), found that the challenges to self-autonomy and independence in managing toilet activity affect participation in CIC among children with myelomeningocele (MMC) and spina bifida. Page and Coetzee (2021) noticed that as a result of the physical disability, access to toilet facilities and other infrastructure while practicing CIC both at schools and homes is affected. In Sudan, Ibrahim et al. (2004) observed that the lack of access to public toilets inhibits the practice of CIC. In Uganda, Bannink et al. (2016b) reported that to ensure independence, parents are required to be available and to closely support children to practice CIC.

**TABLE 1:** Studies included in the review.

Author	Study	Sample or participants	Country	Methodological design	Objectives of the study	Barriers	Facilitators
Bannink Mbazzi et al. 2016	Cognitive abilities of Pre- and Primary School children with spina bifida in Uganda	A total of 133 parents, 133 children with spina bifida and 35 siblings	Uganda	Qualitative semi-structured interviews and quantitative functioning scales measurement	To investigate the cognitive abilities of pre- and primary school children without and with spina bifida in Uganda.	Social barriers towards care and support for children with spina bifida.	Parental support and household income to facilitate travel costs to rehabilitation centres for supplies of catheters.
Orlandin et al. 2020	Difficulties of patients and caregivers in performing clean intermittent catheterisation: A scoping review	-	Brazil	Scoping review following the JBI criteria	To identify the main difficulties reported by patients and caregivers in the use of clean intermittent catheterisation described in the scientific literature.	Difficulty in acquiring materials, adequate place to perform the CIC, routines and overload of the procedure (social nature); menstruation; pain and discomfort while performing the CIC.	No mention of facilitators to practice and usage of CIC.
Kyal et al. 2018	Clean intermittent catheterisation: Barriers and adherence issues in a muslim population	A total of 50 patients with spina bifida, spinal cord injury and other Urinary Tract Infections	Morocco	Prospective, descriptive and analytic study	To analyse barriers and adherence issues of CIC in a muslim arabic population and to present some solutions.	Barriers to CIC include dexterity, positioning challenges, inadequate facilities at work or in public bathrooms, varying types of catheters; cost of supplies, fear and pain felt during catheter insertion.	Self-lubricated disposable catheters with jelly injected into the urethra ease catheterisation and minimise urethral trauma and infection.
Page and Coetzee 2021	South African adolescents living with spina bifida: Contributors and hindrances to well-being	A total of 14 adolescents aged 13–16 years with a primary diagnosis of spina bifida and myelomeningocele and one primary caregiver, either a parent (biological or non-biological) or a legal guardian	South Africa	Explorative qualitative research design and semi-structured interviews	To identify and document the perceptions of adolescents with spina bifida myelomeningocele and their primary caregivers on the factors that contribute to and hinder the well-being of adolescents living with spina bifida myelomeningocele in South Africa.	Limited financial resources inhibited participants’ access to free medical care including access to catheterisation services.	Family support especially the role of caregivers.
Bannink, Idro and Van Hove 2016b	‘I like to play with my friends’: Children with spina bifida and belonging in Uganda	A total 139 families, 139 parents, 97 children with spina bifida and 35 siblings between 4 and 14 years of age	Uganda	Semi-structured interviews utilising the VABS daily functioning and social skills sub-scales	To explore belonging at micro, meso and macro level taking into consideration African childhood disability studies, central concepts of family, cultural conceptions of disability, poverty and the notion of ‘Ubuntu’ and using child friendly culturally adjusted interview methods including play.	Limited financial resources, a lack of transport to rehabilitation centres, and the practice of CIC interfering with the daily functioning of children with spina bifida.	Support of siblings in usage and practice of CIC and in ‘getting around’.
Bannink, Van Hove and Idro 2016c	Parental stress and support of parents of children with spina bifida in Uganda	A total of 134 parents of children with spina bifida	Uganda	Focus group discussions were held with four parent support groups in four different regions in Uganda. The VABS, DFS and PSI/SF	The study aimed to explore the perceived stress and support of parents of children with spina bifida living in Uganda and the factors that influence them.	Practicing catheterisation outside the home and the rehabilitation and the challenges, it comes with.	Parent support system and the support of other household members.
Bannink, Idro and Van Hove 2016a	Family relationships, support, and care: perspectives of children with spina bifida in central Uganda	A total of 60 children with spina bifida and their siblings (30 with spina bifida and 30 of their siblings aged 4 to 14 years)	Uganda	Qualitative research involving semi-structured interviews	To explore family relationships, supports and restrictions, care and inclusion, using the family relations test.	The CIC processes are taking long and hard to navigate among children.	Peer-peer support in learning CIC and support of siblings.
Jeruto et al. 2004	Clean intermittent catheterisation: Overview of results in 194 patients with spina bifida	A total of 416 patients with spina bifida	Kenya	Mixed methods study and data collected included patient demographics, defect type and location, all surgical interventions, all urodynamic and urological assessments	To assess a CIC programme instituted in Kenya and attempt to identify its feasibility and effectiveness.	Economic barriers that affect access to resources necessary for CIC.	Continuous education and training and starting CIC in the newborn period because it is easy to master by parents and is more acceptable to children as they grow up.
Ibrahim, Ibrahim and Enaeema 2004	Effectiveness of educational programme on intermittent catheterisation compliance among non-compliant mothers of spina bifida children at Cheshire Home, Khartoum State, Sudan	A total of 36 mothers of spina bifida children who were not compliant with clean intermittent catheterisation	Sudan	Quasi-experimental study design	To assess the effectiveness of an educational programme on adherence to compliance among non-compliant mothers of spina bifida children.	A lack of access to the public toilet (inadequate shelves or countertops for placing supplies in preparation for CIC), difficult position to insert the catheter, lack of proper training, a lack of community support.	The quality of teaching, supervision, follow-up and catheter availability in the community.
Sims-Williams et al. 2018	Renal outcomes in children with operated spina bifida in Uganda	A total of 65 children with spina bifida aged 10–14 years and their parents or caregivers	Uganda	Quantitative telephone-based assessment study	To determine the extent of renal complications in surviving children with spina bifida.	The lack of (running out of) catheters, a lack of time and the child’s distress (notably those in whom CIC had been initiated at an older age).	Caregivers were trained in the technique of CIC, and a free supply of catheters and the anticholinergic oxybutynin were provided by the IFSBH to families at every visit.

CIC, Clean Intermittent Catheterisation; IFSBH, International Federation of Spina Bifida and Hydrocephalus; JBI, Joanna Briggs Institute; VABS, Vineland Adaptive Behaviour Scales; PSI/SF, Parental Stress Index Short Form; DFS, Daily Functioning Subscales.

#### Theme 5: Limited access to medical care and financial difficulties

Three studies conducted in three countries (1) South Africa (Page & Coetzee 2021), (2) Kenya (Jeruto et al. 2004) and (3) Uganda (Bannink et al. 2016b) reported that limited access to medical care and financial difficulties affect the usage and practice of CIC. Bannink et al. (2016b) found that in Uganda the most stressful part of the daily life of children with spina bifida is finding social spaces and reliable areas to practice CIC. Page and Coetzee (2021) reported that in under-resourced communities in South Africa, limited finances inhibit children with spina bifida from accessing medical care making it hard to practice or use CIC. Similarly, in Kenya, Jeruto et al. (2004) posited that financial constraints hamper the processes to use and practice CIC as simple materials and supplies can be prohibitive in cost for an average family of a child with spina bifida. Jeruto et al. (2004) also observed that catheter and lubricant costs have been a problem for a majority of families in LMIC. The study in Kenya recommended simplifying CIC as much as possible so that economically challenged communities can afford it. In South Africa, Page et al. (2021) reported that the lack of transport to rehabilitation centres to access medical care denies adolescents with spina bifida an opportunity to acquire knowledge and skills in practicing CIC (Page & Coetzee 2021).

#### Theme 6: A lack of facilities to practice Clean Intermittent Catheterisation

Three studies conducted in Uganda (Bannink et al. 2016b, 2016c) and Sudan (Ibrahim et al. 2004) reported a lack of facilities to practice CIC as a hindrance to the uptake of the intervention among children with spina bifida and their families. In Uganda, Bannink et al. (2016c) noticed that the majority of parents practiced catheterisation although they complained practicing it outside the home and the rehabilitation facility was challenging. Bannink et al. (2016c) also noticed that children hardly engaged in CIC and reported that the practice interferes with their daily functioning because of a lack of facilities. In the Ugandan school setting (Bannink et al. 2016b), dirty latrines and a lack of space to practice CIC or even to find water to clean up were challenging. Bannink et al.’s (2016c) findings from Uganda showed that the inability to find alternative facilities to catheterisation outside the home poses a barrier to carrying on the practice and usage in the everyday life of children with spina bifida. While homes were mentioned as better places to practice CIC because of the availability of required resources like water, schools or other places were found to be difficult places to practice CIC because of a lack of resources and privacy (Bannink et al. 2016b).

#### Theme 7: The clean intermittent catheterisation process is long and time-consuming in practice

Three studies conducted in Brazil (Orlandin et al. 2020) and Uganda (Bannink et al. 2016a; Sims-Williams et al. 2018) mentioned that children expressed a concern that CIC processes take a long time and can be hard to navigate. They preferred to use this time to play instead. Sims-Williams et al.’s (2018) findings from Uganda found that children who were initiated into CIC at an older age mentioned the lack of time to practice and use CIC. Orlandin et al.’s (2020) study in Brazil, explored the perception of CIC as a time-consuming procedure. During initial training and practice, patients with spina bifida in Brazil perceived the process as time-consuming based on the time required for mastery of the procedure (Orlandin et al. 2020). Orlandin et al. (2020) observed that the routine of daily practice becomes burdensome to both patients with spina bifida and their parents/caregivers or families.

#### Theme 8: Difficulty in positioning

Two studies conducted in Kenya (Jeruto et al. 2004) and Morocco (Page & Coetzee 2021) mentioned that multiple internal (patient-related) and external factors are barriers to the practice of CIC including physical disabilities encompassing positioning, dexterity and visual impairment to spot the urethra before insertion of the catheter. Jeruto et al.’s (2004) findings from Kenya showed that positioning is a requirement to perform CIC either standing, lying or sitting and yet this is still a limiting factor in the usage and practice of CIC especially among females.

#### Theme 9: A lack of supplies

Ibrahim et al.’s (2004) findings from Sudan showed that the lack of supplies to facilitate adequate toileting practices especially inadequate shelves or countertops for placing supplies in preparation is a hindrance to the practice of CIC (Ibrahim et al. 2004). In Sudan, the inability to access and acquire both local and imported supplies to foster the practice and management of CIC is reported as a barrier to the uptake of the procedure (Ibrahim et al. 2004). Sims-Williams et al.’s (2018) findings from Uganda emphasised that, especially among older children with spina bifida, the practice of CIC was abandoned because of a lack of catheters and distress.

#### Theme 10: Perception of self, support, and care

In Uganda, Bannink et al. (2016a) reported that children with spina bifida were concerned in terms of perception of self, support and care with the processes of managing CIC even when their caregivers encouraged them to perform CIC independently. While there were barriers to the usage and practice of CIC expounded in this section, there were also facilitators to the usage and practice of the procedure discussed in detail in the next section of this review.

### Facilitators to use and practice clean intermittent catheterisation

The major themes identified as facilitators to usage and practice of CIC included tolerance of infants in the uptake of CIC fostering long-term adherence, the support from family and community members, quality of training and benefits of continuous practice of CIC for long-term uptake and adherence. Other facilitators included the utilisation of lubricants, reuse of catheters and other affordable materials to families available in the local communities and rehabilitation centre, family, peer and community support, availability of sufficient finances to access care, the neonatal institution of CIC and follow-up of healthcare provision by rehabilitation workers. These themes are presented next.

#### Theme 1: Tolerance of infants in uptake of clean intermittent catheterisation

Kyal et al.’s (2018) study from Morocco observed that initiating catheterisation in infancy has been identified by caregivers as an ease of learning and adjustment. Infants are more tolerant and technically easier to catheterise, unlike toddlers who are behaviourally more challenging because of their growth, development and self-awareness (Kyal et al. 2018). The review also reports that in Morocco, educating rehabilitation workers regarding CIC facilitates their ability to concentrate and administer care for children based on set protocols. In Morocco, it is further noticed that to facilitate adjustment to CIC, it is important to utilise teaching methods focusing on the developmental and social maturity of a particular age or age group of children at the level of their understanding of the CIC learning process (Kyal et al. 2018).

#### Theme 2: Support from family and community members

A study conducted in Uganda mentioned that sometimes when children with spina bifida in the school or community were challenged in the use of CIC, they requested assistance from teachers or parents (Bannink Mbazzi et al. 2016). Children with spina bifida are open to accessing support from individuals they trust and those who can help (Bannink Mbazzi et al. 2016). While it is considered difficult for patients to incorporate intermittent catheterisation in their daily lives in Uganda, adjustment of social life and encouragement to practice CIC at home facilitates the practice of CIC as often patients with spina bifida are reminded of bladder emptying (Bannink Mbazzi et al. 2016). Page et al. (2019) found that in South Africa family support is an instrumental facilitator to the practice of CIC among adolescents with spina bifida (Page & Coetzee 2021). The need for consistent attention from caregivers facilitates the correct usage and practice of CIC (Page & Coetzee 2021). Besides, quality family time fosters the desire to gain knowledge and share experiences of practicing CIC (Page & Coetzee 2021). According to Page et al. (2021) biological mothers are the pillars of guidance to children or adolescents practicing CIC (Page & Coetzee 2021).

Bannink et al. (2016c) in their findings from Uganda mentioned that the parent support system has a significant influence on the practice of CIC. In Uganda, the support of another adult in the household in the family to care for a child with spina bifida is instrumental in ensuring regular practice and usage of CIC (Bannink et al. 2016c). This support system has a significant contribution to improving parent–child interaction that extends to a lifelong understanding of the need to catheterise (Bannink et al. 2016c). Similarly, one of the studies also conducted in Uganda by Bannink et al. (2016) and colleagues revealed that support from siblings across a range of activities bolsters their confidence to practice CIC and learn over time. Bannink et al. (2016a) report that the peer-to-peer practice of CIC encourages participation and instils confidence in children with encouraging results in continence management. In Brazil, Orlandin et al. (2020) posited that psychological support and regular follow-up of children and their families practicing CIC are facilitators of long-term adherence.

#### Theme 3: Training, quality of training and benefits of continuous practice of Clean Intermittent Catheterisation

Through findings from Uganda, Bannink et al. (2016) noticed that children who continuously practiced CIC had increased participation in daily activities both at home and in school. Bannink et al. (2016c) found that the quality of the training environment for rehabilitation workers, parents and patients is a facilitator of the usage and practice of CIC. Page and Coetzee (2021) in South Africa highlight that patients with an overactive bladder were familiar with intermittent catheterisation after a few weeks of training. In South Africa, the basis for their motivation to understand the CIC management process is anchored on the expected results of bladder emptying (Page & Coetzee 2021). In Sudan, Ibrahim et al. (2004) observed that the frequency of CIC training is determined by the patient’s ability to gain confidence in self-managing CIC. The facilitators to usage and practice include the quality of teaching, supervision and follow-up in the community (Ibrahim et al. 2004). In Uganda, Sims-Williams et al. (2018) reported that the provision of CIC training to children with spina bifida and their caregivers facilitates usage and practice (Sims-Williams et al. 2018).

Orlandin et al.’s (2020) findings from Brazil showed that with practice and increasing confidence in CIC usage, children with spina bifida may not require much more time than normal to pass urine. Orlandin et al. (2020) also noticed that spina bifida patients in Brazil were reported to be motivated to uptake CIC once they knew the process would be long-term and they were positive on prospective outcomes of improving bladder functioning. In Brazil, it is recommended that while providing training to parents and caregivers to facilitate the acquisition of knowledge and skills in managing CIC, instructors should evaluate patients’ conditions and provide customised education (Orlandin et al. 2020). Among the Brazilian children with spina bifida and their parents, the desire to achieve independence in dressing, bathing, toileting and skin care while practicing CIC facilitates autonomy and a sense of belonging (Orlandin et al. 2020).

#### Theme 4: Utilisation of lubricants, reuse of catheters and other cost-effective materials

Orlandin et al. (2020) in a study with a Brazilian population reported that lidocaine gel lubricant is widely used to lubricate the urethra and it is effective in reducing pain and sensitivity. In Brazil, to reduce the risk of urethral trauma, promoting some urethral dilation and facilitating the introduction of the urinary catheter have proven beneficial (Orlandin et al. 2020). Orlandin et al. (2020) reported that to facilitate the reuse of urinary catheters and promote hygiene, several techniques are reported in multiple literature including putting the urinary catheter in boiling water, washing it in running water, storing it in a solution with vinegar, detergent or 70% alcohol or heating it in the microwave. In Sudan, Ibrahim et al. (2004) observed that the facilitators of usage are also premised on catheter availability in the community. Similarly, Bannink et al. (2016) reporting findings from Uganda noticed that private donors funded CIC and bowel management trainings including the provision of low-cost materials and follow-up of children with spina bifida at home.

#### Theme 5: Provision of free supplies after training

In Uganda, caregivers who received training were provided a free supply of catheters and anticholinergic oxybutynin at every visit (Sims-Williams et al. 2018) fostering their support to practice of CIC among their children in the communities. Sims-Williams et al. (2018) reported anticholinergic oxybutynin was administered intravenously, thus motivating caregivers and children with spina bifida to use and practice CIC (Sims-Williams et al. 2018).

#### Theme 6: Neonatal institution of clean intermittent catheterisation and the follow-up of healthcare provision by rehabilitation workers

Jeruto et al.’s (2004) research from the Kenyan population recommended starting CIC in the new-born period as it is easier to master by parents and is more acceptable to children as they grow. This study also points out that an aggressive approach with the immediate neonatal institution of CIC has evidence of reducing the need for surgical procedures later in life (Jeruto et al. 2004). Jeruto et al. (2004) affirm that overcoming significant barriers and further parental education is needed to offer infants with spina bifida a good standard of care. In terms of healthcare provision by rehabilitation workers, Bannink et al. (2016b) in their study from Uganda noticed that the distinction between routine general health visits and visits to occupational or physiotherapists enables children to understand the role rehabilitation workers have in their lives. In Uganda, Bannink et al. (2016b) also found out that rehabilitation workers foster routine usage and practice of CIC whenever they conduct home visits.

#### Theme 7: Availability of sufficient finances

In Uganda, household income and overall parental support were reported as important key predictors to continence management (Bannink Mbazzi et al. 2016). In families with higher incomes, transport costs to rehabilitation centres is easily met to access supplies. In this case, parents are able to support their children to carry out CIC several times in a day leading to improved quality of life, earlier better motor function and sufficient continence management (Bannink Mbazzi et al. 2016).

## Discussion

Our study highlights the results of 10 studies reviewed on the barriers and facilitators in practice and usage of the CIC among children with spina bifida and their families. While some of the barriers are manageable at the family and societal level, others are manageable at rehabilitation units and hospitals. The barriers include fear, pain and discomfort during catheter insertion, and a lack of quality training from rehabilitation experts for children, parents and caregivers. Other barriers include challenges to self-autonomy and independence in managing toilet activity both at home and in schools. The articles also mentioned limited access to medical and rehabilitation care and financial difficulties, a lack of facilities to practice CIC, the long and time-consuming processes of conducting CIC, difficulty in positioning, a lack of supplies and a negative perception of self, support and care. To counter these barriers, facilitators from our review included the tolerance of infants in uptake of CIC, support from family and community members. The provision of training, quality of training and benefits of continuous practice of CIC also support CIC. The utilisation of lubricants, reuse of catheters and other cost-effective materials facilitates usage and practice. Other facilitators included neonatal institution of CIC and provision of quality training, follow-up of healthcare provision by rehabilitation workers and lastly the availability of sufficient finances.

Our study found out that difficulty in positioning affects the detection of the urethra before insertion of the catheter and yet positioning facilitates the practice of CIC. Bolinger and Engberg (2013) in a similar study conducted in the United States found that in females especially, positioning is a limiting factor, and often females require consistent practice with a patient trainer to enable them to grasp the key steps and processes in conducting CIC. Similarly, in the United States, Bauer et al. (2023) postulated that other than physical barriers, psychological factors also affect adherence to usage and practice of catheterisation. These factors include misconceptions, fear and stigma of using a catheter to empty urine leading to infections.

Our study shows that outside the rehabilitation units, family, peer and community support are instrumental in facilitating the usage and practice of CIC, and that the follow-up by rehabilitation workers strengthens the support to children with spina bifida. These results are inconsistent with the findings from a study conducted in a school environment in the United Kingdom mentioning that there are no regular or local policies for CIC, and overtime school staff, parents and health practitioners have experienced feelings of anxiety, concern and inadequacy because of a lack of knowledge on the programmatic steps to conduct CIC (Fishwick & Gormley 2004). In the absence of strong policies to comprehensively manage CIC, children’s school attendance, achievement and socialisation are affected. If there is limited rehabilitation staff knowledge and understanding of bladder problems, support to children in the school and environment is affected (Fishwick & Gormley 2004).

Our findings posit that the availability of cost-effective materials and reusable items facilitates the use and practice of CIC. Previous studies have shown that the reusable aspect of low cost materials with or without lubricants facilitates practice for children with spina bifida irrespective of the scientific knowledge behind the technique (Bogaert et al. 2004; Mazzo et al. 2017). Our study found that practicing CIC is time-consuming and thus affects participation in other daily activities of life. In a related study conducted in Uganda, it is further noticed that the children experienced extensive urine leakage and were worried that they would miss out on leisure time in school as a result of failure to regulate the time and frequency of toilet visits affecting their practice of CIC (Bannink et al. 2015). Our study reveals that the practice of CIC interferes with the daily functioning of children and parents are often required to be close to children at home, community and at the rehabilitation centres as monitors.

Contrary to grey literature from IFSBH (Bauwens 2014), the review did not show any negative perceptions of illustrations and pictorials used during CIC trainings or negative responses from parents on the use of CIC.

In addition, sociocultural perceptions, myths and misconceptions, stigma and discrimination, and improper handling of catheters as listed by IFSBH (Bauwens 2014) are likely to play role but did not come out strongly from our literature review.

In our review it was found that it is important to initiate CIC at neonatal stages (from birth to 4 weeks) as it is easier to master by parents and it becomes more acceptable to children as they grow. The finding of the study on the neonatal institution of CIC underpins the fact that infants are more tolerant and technically easier to catheterise and it is important to start CIC as early as possible. This is subsequently a prerequisite to save the kidneys of new-borns. In a similar study, it is mentioned that it is important to consider age and other factors while initiating CIC (Herbert, Welk & Elliott 2023). It is expressed that age alone is not an internal barrier that children with spina bifida face but rather children between 2 and 17 years of age were able to self-catheterise; others even performed CIC independently at the age of 9 (Herbert et al. 2023). Similar findings also underpin that spina bifida patients were reported to be motivated to uptake CIC once they knew the process would be long-term and they were positive on prospective outcomes of improving bladder functioning (Cobussen-Boekhorst et al. 2016). The basis for their motivation to understand the CIC management process is anchored on the expected results of bladder emptying (Cobussen-Boekhorst et al. 2016).

None of the studies in this scoping review mentioned aspects around post-discharge management after surgeries or after attending services at rehabilitation centres and yet Jaquet et al. (2009) postulate that there is evidence of poor follow-up of set appointments and instructions by families and caregivers as required by the health or rehabilitation workers and patients who access CIC services. Other studies reason that families and patients with spina bifida experience crisis reactions adversely affecting their performance especially after being informed of the news of potentially having to carry CIC as a lifelong process (Jaquet et al. 2009). To manage post-discharge challenges, CIC is recommended to be carried out in the presence of a caregiver without which, there is difficulty in the follow-up of the practice and usage of CIC especially when children with spina bifida are in environments outside the home such as at school (Lim et al. 2016; Vaidyanathan et al. 2011).

## Limitations

While the team searched the literature exhaustively within the scope and objectives of the study, there may have been further unpublished grey literature that we may have not included. Country reports and other grey literature could have provided insights and evidence on barriers and facilitators but the search was limited to hand-search of the initially included sources such as PubMed, SCOPUS or Google Scholar. We also note that the study protocol was not registered and is a limitation, but we do understand that reviews that commence with a detailed protocol, registered or not, do meet the requirements of reporting bias and transparency. We also limited our search to studies published in English and may have missed other relevant articles for inclusion. The literature search period could have also limited access to other relevant publications by the objectives of the scoping review. With the network created in the management of CIC, experts from rehabilitation partners (majorly funded by Child-Help International) were consulted. We consulted experts including neurologists, occupational therapists, urologists, orthopaedic surgeons and social workers. While this is so, these experts were not involved in review of selected articles. We believe their input could have been valuable in providing insights and recommendations on the topic.

## Recommendations

### Training and capacity building

This review shows that training and capacity building provided by rehabilitation workers on catheterisation to both children and caregivers is instrumental in ensuring continence. The knowledge and understanding of caregivers, children and parents are centre-stage in ensuring the uptake of the practice of CIC. The rehabilitation workers should provide training to children with spina bifida, acclimatise them to the daily routines of practicing CIC and provide age-appropriate rationale for the need to practice CIC as they grow. In order to strengthen the management of CIC, it is important to coordinate and monitor family, peer and community support for effective implementation. Overall, lobbying for the provision of supplies, training and finances is fundamental in providing children assurance of long-term management of incontinence.

### Utilisation of cost-effective materials

In practical terms, the utilisation of cost-effective materials and reusable items in low-income countries provides alternative solutions that promote continence in children with spina bifida. It is recommended to initiate CIC at neonatal stages (from birth to 4 weeks) so parents can carry on the process and initiate their children to CIC as they grow.

### Strengthening community-based rehabilitation and community-based inclusive development initiatives

The rehabilitation centres should strengthen Community Based Rehabilitation (CBR) initiatives geared towards empowering children with spina bifida and their families to overcome physical and sociological barriers in the communities in which they thrive. Community Based Inclusive Development (CBID) on the other hand should be strengthened so that children with spina bifida and their families make informed decisions on practicing and the implications it has on continence management and ultimately kidney saving. In the global discourse, the study recommends positioning challenges, barriers or inhabitants to functioning and health among persons with disabilities as a priority for advocacy for holistic support. The World Health Organization and government ministries in LMICs should rally support towards providing easily accessible information including the limitations children with spina bifida and their families face. A global agenda towards management of NTDs with significant efforts in managing the barriers to the practice of CIC is paramount in fostering holistic functioning among children with spina bifida.

### Collaboration among stakeholders and encouraging early initiation of CIC among children

The review recommends collaboration with healthcare providers, families and support networks to pioneer strategies to streamline CIC practice both at home and in the community. We do recommend that the early initiation of children in CIC instils confidence, fosters independence and improves long-term health outcomes.

## Conclusion

The research evidence in low-income countries on the barriers and facilitators of the CIC explored in this review presents the gaps and opportunities to improve the management of CIC in children with spina bifida and their families. Within the family, children can be supported to practice CIC even if physical and psychological barriers are at play. The rehabilitation practitioners, children and caregivers can interweave knowledge acquisition, usage and practice of CIC by embracing the usage of low-cost materials to facilitate bladder emptying. The role that continuous training and practice plays is fundamental in encouragement of long-term adherence to CIC and other continence management practices. It is also beneficial to utilise simple and practical steps in managing CIC while providing trainings.
